# Interactions between the circadian clock and TGF-β signaling pathway in zebrafish

**DOI:** 10.1371/journal.pone.0199777

**Published:** 2018-06-25

**Authors:** Hadas E. Sloin, Gennaro Ruggiero, Amir Rubinstein, Sima Smadja Storz, Nicholas S. Foulkes, Yoav Gothilf

**Affiliations:** 1 School of Neurobiology, Biochemistry and Biophysics, George S. Wise Faculty of Life Sciences, Tel Aviv University, Tel Aviv, Israel; 2 Sagol School of Neuroscience, Tel Aviv University, Tel Aviv, Israel; 3 Institute of Toxicology and Genetics, Karlsruhe Institute of Technology, Eggenstein, Germany; 4 Blavatnik School of Computer Science, Tel Aviv University, Tel Aviv, Israel; University of Lübeck, GERMANY

## Abstract

**Background:**

TGF-β signaling is a cellular pathway that functions in most cells and has been shown to play a role in multiple processes, such as the immune response, cell differentiation and proliferation. Recent evidence suggests a possible interaction between TGF-β signaling and the molecular circadian oscillator. The current study aims to characterize this interaction in the zebrafish at the molecular and behavioral levels, taking advantage of the early development of a functional circadian clock and the availability of light-entrainable clock-containing cell lines.

**Results:**

*Smad3a*, a TGF-β signaling-related gene, exhibited a circadian expression pattern throughout the brain of zebrafish larvae. Both pharmacological inhibition and indirect activation of TGF-β signaling in zebrafish Pac-2 cells caused a concentration dependent disruption of rhythmic promoter activity of the core clock gene *Per1b*. Inhibition of TGF-β signaling in intact zebrafish larvae caused a phase delay in the rhythmic expression of *Per1b* mRNA. TGF-β inhibition also reversibly disrupted, phase delayed and increased the period of circadian rhythms of locomotor activity in zebrafish larvae.

**Conclusions:**

The current research provides evidence for an interaction between the TGF-β signaling pathway and the circadian clock system at the molecular and behavioral levels, and points to the importance of TGF-β signaling for normal circadian clock function. Future examination of this interaction should contribute to a better understanding of its underlying mechanisms and its influence on a variety of cellular processes including the cell cycle, with possible implications for cancer development and progression.

## Introduction

As a result of the earth's rotation around its axis, the majority of organisms are exposed to rhythmic daily changes in their environment, including illumination, ambient temperature and food availability. Organisms adapt to these changes by exhibiting a wide variety of physiological and behavioral daily rhythms which are driven by an intrinsic timing mechanism known as the circadian clock. The underlying mechanism of the circadian clock relies on a network of positive and negative transcriptional-translational feedback loops, which constitute a molecular oscillator that drives rhythmic expression of clock components with a period of approximately 24 hr. In vertebrates, positive elements of this feedback loop include the proteins Clock and Bmal1, which heterodimerize and act as transcription factors for genes containing E-box enhancer elements, including the genes encoding negative elements of the feedback loop, namely *Per* and *Cry*. After being translated, Per and Cry proteins heterodimerize, enter the nucleus and suppress the activity of Clock:Bmal1, thus downregulating their own transcription. A new cycle begins when Clock:Bmal1 repression is eliminated via the degradation of Per and Cry. This core molecular mechanism affects other cellular functions by directing the circadian expression pattern of a variety of genes, collectively called clock-controlled genes (CCGs), which in turn regulate downstream processes. Some of these CCGs, for example *Dec1* and *Rev-erbα*, feedback on the core clock mechanism itself [1–4].

The circadian clock influences nearly all aspects of an organism’s physiology and behavior, such as sleep-wake cycles, changes in body temperature, hormone secretion and metabolism [5,6]. Disruption of the circadian clock system and dis-synchronization of its derived rhythms have been suggested to increase the risk for several diseases and syndromes, including tumorigenesis and tumor progression [7,8], metabolic syndromes and obesity [9], as well as Alzheimer’s disease [10,11]. However, although the molecular mechanism of the circadian clock is well characterized and the influence of the clock on multiple physiological processes has been well documented, the underlying mechanisms linking clock disruption with these disorders are not fully understood.

TGF-β is a widely expressed and secreted protein that has been shown to play a key role in multiple processes, including the immune response, cell differentiation and proliferation [12], and has been particularly well studied in the context of cancer biology [13]. The binding of TGF-β to one of its receptors, ALK4, ALK5 or ALK7, leads to the phosphorylation of Smad2 or Smad3, their association with Smad4, and their translocation into the nucleus. In the nucleus, the Smad2/3-Smad4 complex act as a transcription factor, in association with various co-activators and co-repressors to activate or repress the transcription of many genes and cellular processes [14–17]. TGF-β is associated with elaborate negative feedback mechanisms. These mechanisms include inhibitory SMADs (like SMAD7) and co-repressors (such as TGIF1 [14,18]). An effect of TGF-β signaling on the circadian clock was initially proposed based on evidence that activation of ALK receptors by TGF-β leads to the induction of Dec1 activity and consequent resetting of the molecular oscillator [19]. This evidence was later reinforced by studies revealing that TGF-β2 inhibits the expression of several clock genes [20]. Moreover, it was shown that *Smad3* mRNA exhibits rhythmic expression in human cell lines and the mouse liver [21], and that TGF-β and phosphorylated Smad3 (pSmad3) proteins exhibit a circadian expression pattern in the hypothalamic superchiasmatic nucleus, the site of the master clock in mammals [22]. Together, these recent findings suggest a bi-directional interaction between the circadian clock and TGF-β signaling. Further examination of this interaction should shed light on important processes known to be regulated by both systems, including cell cycle, cancer development and progression, as well as other physiological processes.

Here, we have used the zebrafish as a model to characterize in more detail the functional links between TGF-β signaling and the circadian clock. We demonstrate that a bidirectional interaction between the clock and TGF-β signaling pathways exists at both the molecular and behavioral levels. Importantly, we reveal that TGF-β signaling is essential for normal circadian clock function in this species.

## Results

### 1. Circadian clock-regulated components of the TGF-β signaling pathway

#### 1.1 TGF-β signaling genes exhibit clock-dependent circadian expression pattern in the zebrafish

To explore the influence of the circadian clock on TGF-β signaling, we first tested whether TGF-β signaling-related genes exhibit a circadian expression pattern in the zebrafish. First, we analyzed transcriptome data from a previous microarray experiment performed using whole zebrafish larvae, which revealed 2,847 genes showing a circadian expression pattern [23]. Examination of this data revealed that TGF-β signaling genes *Smad3a*, *Tgif1* and *Smad7* exhibit rhythmic expression in zebrafish larvae. *Smad3a* displays high levels at the end of the night and at the beginning of the light period, peaking at CT4, and then low levels at the beginning of the night. Instead, *Smad7* and *Tgif1* display high levels at the beginning of the night, with a peak at CT12, and low levels at the beginning of the light period. The expression of other TGF-β related genes, such as *tgfb1a*, *tgfb2*, *tgfb3* and *smad3b* did not show significant circadian rhythmicity.

**Fig 1:**
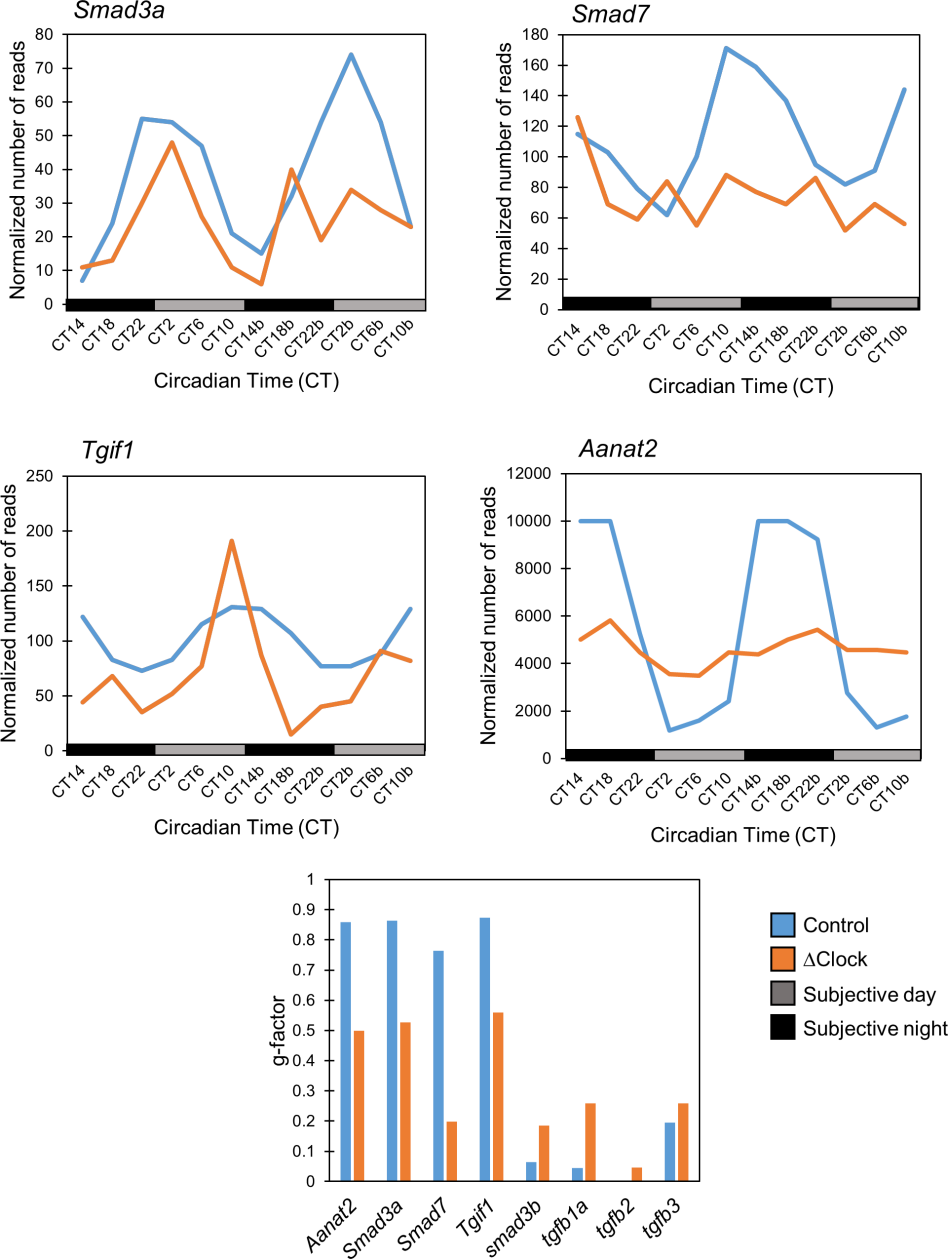
Clock-dependent circadian expression pattern of *Smad3a*, *Smad7*, and *Tgif1* in the adult zebrafish pineal gland. Data mining of a previous RNA-sequencing experiment reveals that the TGF-β signaling genes *Smad3a* (top left), *Smad7* (top right) and *Tgif1* (mid left) exhibit a circadian expression pattern in the zebrafish pineal gland under constant darkness (DD), which is diminished in transgenic fish with a disrupted pineal circadian clock, Tg(*aanat2*: EGFP-ΔCLK), similarly to the known clock-controlled gene, *aanat2* (mid right). Grey bars represent subjective day and black bars represent subjective night. CT0 corresponds to “subjective lights on”, CT12 to “subjective lights-off”. *Bottom*: a comparison between the g-factor values of different TGF-β signaling genes in both control and transgenic fish. In control fish, *Smad3a*, *Smad7* and *Tgif1* exhibit rhythmic expression patterns with a high g-factor value, similar to that of *aanat2*, indicating that they indeed exhibit a circadian expression pattern. In ΔClock fish these genes lose their rhythmic expression pattern as indicated by significantly lower g-factor values, implying that their circadian pattern is regulated by the core mechanism of the circadian clock. The TGF-β related genes *Tfgb1a*, *Tgfb2*, *Tgfb3* and *Smad3b* exhibit low g-factor values in both groups, indicating they are not expressed in a circadian manner in the zebrafish pineal gland.

We next tested whether these genes also exhibit a circadian expression pattern in adult zebrafish. We initially analyzed existing microarray data obtained from adult zebrafish brains, which demonstrated 714 genes that exhibit a circadian expression pattern [24]. Examination of this data revealed that while *Smad3a* exhibits a circadian expression pattern in the adult zebrafish brain, peaking at CT4, *Smad7*, *Tgif1* and other TGF-β related genes do not exhibit such oscillations. We then examined RNAseq data obtained specifically from the pineal gland of adult zebrafish, which is considered to play a key role in coordinating circadian rhythmicity in the entire organism [4,25]. Amongst 308 genes which exhibited a circadian expression pattern in the zebrafish pineal gland [23,24], the TGF-β signaling genes *Smad3a*, *Tgif1* and *Smad7* exhibit a rhythmic expression, peaking at CT2, CT10 and CT10, respectively (Fig 1). The extent to which the circadian rhythmicity of each gene corresponded to a period of 24 hr was quantified using a 'g-factor' value. To calculate the 'g-factor', the gene expression data, which is a time dependent signal, is converted into a frequency dependent signal using the Fast Fourier Transform (FFT). The ratio between the power of frequency that corresponds to the 24 hr period to the sum of powers of all frequencies is called the 'g-factor', and its values range between zero and one [26,27]. The 'g-factor' of the three TGF-β signaling genes was similar to that of known clock-controlled genes, such as *aanat2*. Importantly, as evident in the ‘g-factor’ values, circadian rhythmicity in the expression of these three genes was diminished in the transgenic zebrafish line Tg(*aanat2*:EGFP-ΔCLK) which expresses a dominant-negative form of CLOCK in the melatonin-producing photoreceptor cells of the pineal gland, thereby disrupting circadian-clock function [28]. This observation suggests that rhythmic expression of these TGF-β signaling genes is driven, directly or indirectly, by the core molecular clock in the pineal.

#### 1.2 *Smad3a* mRNA shows a circadian clock-controlled expression in zebrafish larvae heads

**Fig 2:**
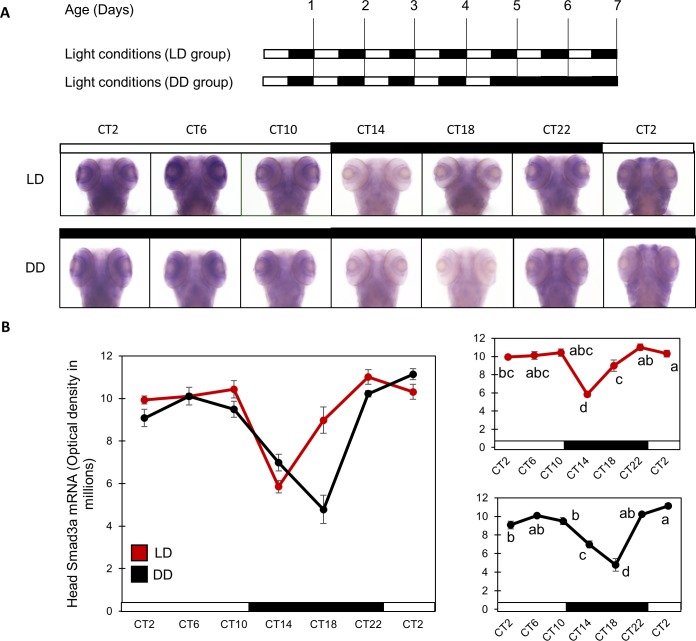
*Smad3a* mRNA shows a circadian clock-controlled expression in zebrafish larvae heads. *Smad3a* mRNA exhibits a circadian rhythm expression pattern in zebrafish larvae heads, with mRNA expression significantly affected by sampling time (*p<00*.*1*, two-way ANOVA), showing higher expression levels during late night-time and daytime than early night-time. This pattern persists in constant darkness, suggesting that it is regulated by the circadian clock. In addition, *Smad3a* mRNA expression is also significantly affected by lighting conditions (*p<0*.*001*, two-way ANOVA), with a significant interaction between sampling time and light conditions (*p<0*.*001*, two-way ANOVA) (n = 15/group). (A) Top panel: schematic representation of the experimental design. The horizontal bars represent the lighting conditions before and during sampling; white boxes represent light and black boxes represent dark periods. Bottom panel: whole mount ISH images for *Smad3a* mRNA (dorsal views) of representative specimens raised under LD cycles until and during sampling or kept under DD during sampling. Circadian times are indicated for each sample. CT0 corresponds to “subjective lights on”, CT12 to “subjective lights-off”. White bars represent light phases and black bars represent dark phases. (B) Left: quantification of signal intensities in the head of larvae under LD and DD. Values represent the mean ±SE optical densities of the head signals. White bars represent subjective day and black bars represent subjective night. Right: Different letters represent statistically different values within each photoperiodic treatment (*p<0*.*05*, one-way ANOVA, Tukey’s test). This experiment was repeated twice resulting in a similar outcome. The represented results are of one experiment.

Next, we investigated the spatio-temporal expression pattern of *Smad3a* in the whole organism, using whole mount *in situ* hybridization (ISH). Zebrafish larvae were exposed to 12 hr:12 hr light/dark (LD) cycles for 5 days, and on the night of the 5^th^ day of development, half were transferred to DD. During the 6^th^ and 7^th^ days of development the larvae were collected at 4 hour intervals and mRNA was detected and semi-quantified by a whole mount ISH protocol (n = 15/group). This analysis indicated that *Smad3a* mRNA exhibits a circadian expression pattern in the zebrafish larva head (Fig 2). The observed levels of *Smad3a* mRNA expression was significantly affected by sampling time (*p<0*.*001*, two-way ANOVA), showing higher expression levels during late night-time and daytime than early night-time. This pattern persisted in DD, indicating that it is regulated by an endogenous circadian clock. *Smad3a* mRNA expression was also significantly affected by lighting conditions (*p<0*.*001*, two-way ANOVA), with a significant interaction between sampling time and light conditions (*p<0*.*001*, two-way ANOVA). The rhythmic expression pattern of *Smad3a* under DD in the zebrafish larva head was similar to that found in our previous transcriptome analyses (See section 1.1). In contrast to *Smad3a*, another zebrafish paralog of *Smad3*, *Smad3b*, did not show any time- or light-dependent expression pattern (S1 Fig), corroborating the results of previous transcriptome analyses (See section 1.1). These results sustain the notion that *Smad3a* exhibits a circadian expression pattern regulated by the circadian clock in the whole larva head.

### 2. TGF-β signaling pathway affects the circadian clock

#### 2.1 Disruption of TGF-β signaling interferes with the molecular circadian clock in zebrafish PAC-2 cells

Previous studies have demonstrated that TGF-β influences the expression of several clock genes in human cell lines and mice liver [21]. In order to more precisely examine the influence of TGF-β signaling on peripheral circadian clock function, we tested the effect of pharmacologically blocking TGF-β signaling on the molecular circadian oscillator in zebrafish PAC-2 cells stably transfected with a clock gene promotor-reporter construct, Tg(-3.1)per1b::luc [29].

**Fig 3:**
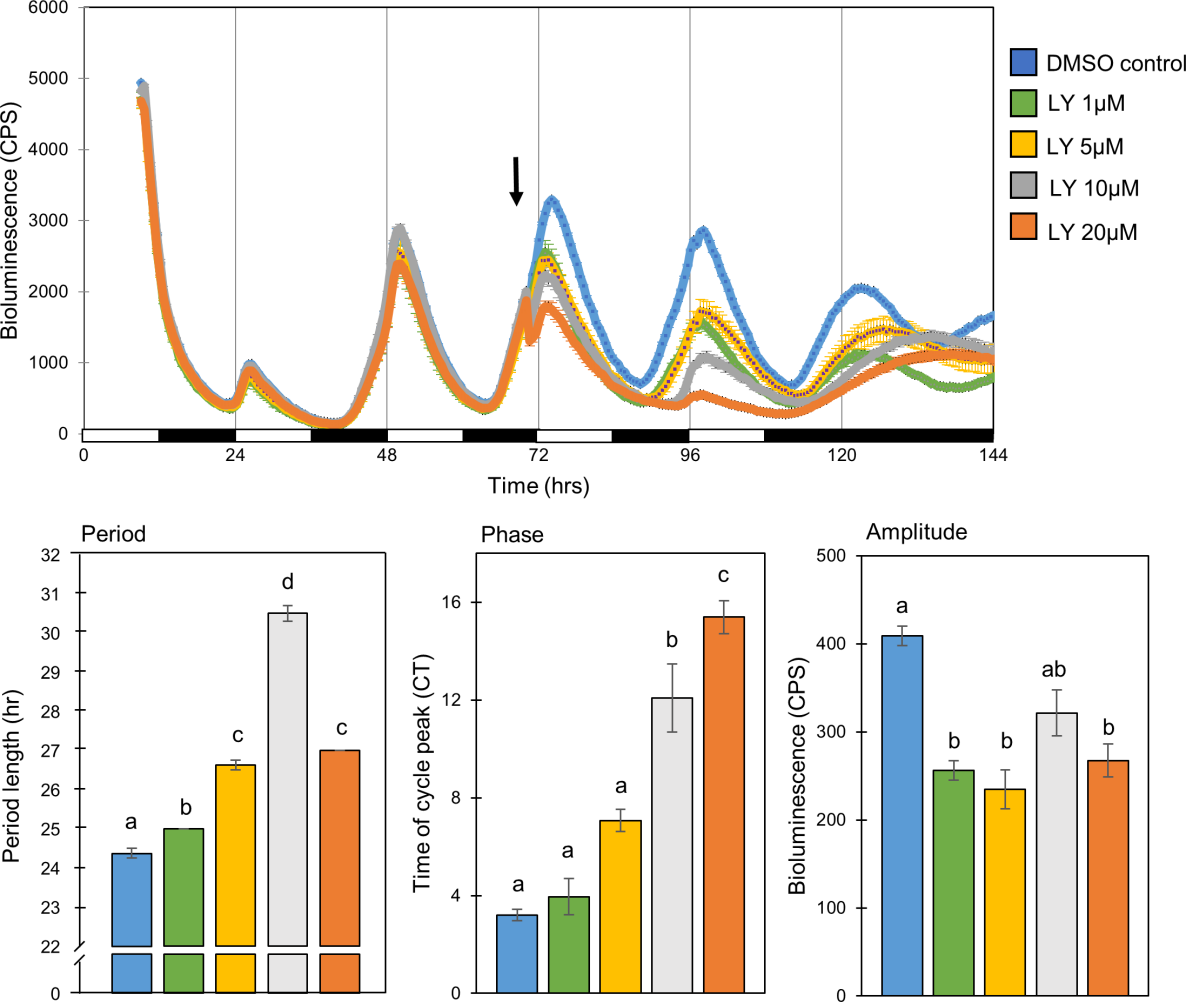
The molecular circadian oscillator in PAC-2 cells is significantly altered by TGF-β inhibition. Rhythmic *Per1b* promotor activity in the zebrafish PAC-2 cell line was significantly altered by the addition of the TGF-β inhibitor LY-364947 in a dose-dependent manner in comparison to DMSO treated control (n = 4-12/group). *Upper panel*: Luciferase bioluminescence, driven by the *per1b* promotor, is plotted on the y-axis and time (hours) on the x-axis. The horizontal bars represent the lighting conditions during the measurements; white boxes represent light periods and black boxes represent dark periods. *Lower panel*: cells which were exposed to LY-364947 exhibit rhythms of longer period (*p<0*.*001*, one-way ANOVA), a phase delay (*p<0*.*001*, one-way ANOVA), and a lower amplitude of expression (*p<0*.*001*, one-way ANOVA).Different letters represent statistically different values within each parameter (*p<0*.*05*, Tukey’s test). This experiment was repeated twice, resulting in comparable results. The represented results are of one experiment.

Cells were exposed to 3 LD cycles for entrainment. Then, 30 minutes before lights on a selective ATP-competitive inhibitor of the TGF-β receptor ALK5, LY-364947, was added to the cell culture medium at different concentrations (1, 5, 10, 20 μM). This inhibitor was previously shown to inhibit TGF-β-Smad3 mediated signaling in zebrafish larvae [30]. Cells were maintained in LD for an additional 2 days and then transferred to DD for 3 additional days. Luciferase activity was monitored and compared with that of vehicle treated control cells (n = 4/group). The addition of TGF- β inhibitor LY-364947 altered the clock controlled rhythmic activity of the *per1b* promotor in a dose-dependent manner (Fig 3).

Treatment with TGF-β inhibitor LY-364947 led to a significant lengthening of the period of *Per1b* promotor activity in all inhibitor concentrations (25±0, 26.62±0.12, 30.5±0.2, 27±0.0 hr for 1, 5, 10 and 20 μM, respectively, compared to 24.37±0.12 for the DMSO treated control, *p<0*.*001*, one-way ANOVA, *p<0*.*05*, Tukey's post-hoc). This led to a significant dose-dependent phase delay in higher (10 and 20 μM) inhibitor concentrations (the time of the first peak after the cells were transferred to DD was at CT 3.95±0.74, 7.08±0.46, 12.08±1.4 and 15.4±0.67 for 1, 5, 10 and 20 μM, respectively, compared to CT 3.2±0.24 for the DMSO treated control, *p<0*.*001*, one-way ANOVA, *p<0*.*05*, Tukey's post-hoc). The treatment also led to a reduction in the amplitude of rhythmic *Per1b* promotor activity during the first DD cycle after exposure in all concentrations but 10μM (256.25±20.51, 234.62±22.38, 321.5±26.12, 267.33±18.78 CPS for 1, 5, 10 and 20 μM, respectively, compared to 409±11.04 for the DMSO treated control, *p<0*.*001*, one-way ANOVA, *p<0*.*005*, Tukey's post-hoc). An additional experiment was performed using an alternative TGF-β inhibitor of both TGF-β receptors ALK-4 and ALK-5, SB-505124. Inhibition with SB-505124 resulted in very similar effects on amplitude and period of *per1b* promotor activity, but with insignificant effects on phase (S2 Fig).

These results indicate that TGF- β signaling is essential for the rhythmic promotor activity of a key clock gene in the PAC-2 zebrafish cell line, and therefore demonstrate the importance of TGF-β signaling for normal function of the circadian clock mechanism.

In order to further demonstrate the influence of the TGF-β signaling system on the molecular circadian clock, we tested the effects of TGF-β signaling activation on the molecular circadian oscillator in PAC-2 cells. This was done by applying the compound Alantolactone, which disrupts Cripto-1/ActRII complexes resulting in an indirect induction of activin/Smad3 signaling [31]. Cells were exposed to 3 LD cycles for entrainment. Then, at CT 23.5 Alantolactone was added to the cell culture medium at different concentrations (1, 5 and 10 μM, n = 8/group) and cells were transferred to DD for additional 3 days. Luciferase activity was monitored and compared with that of vehicle-treated control cells.

**Fig 4:**
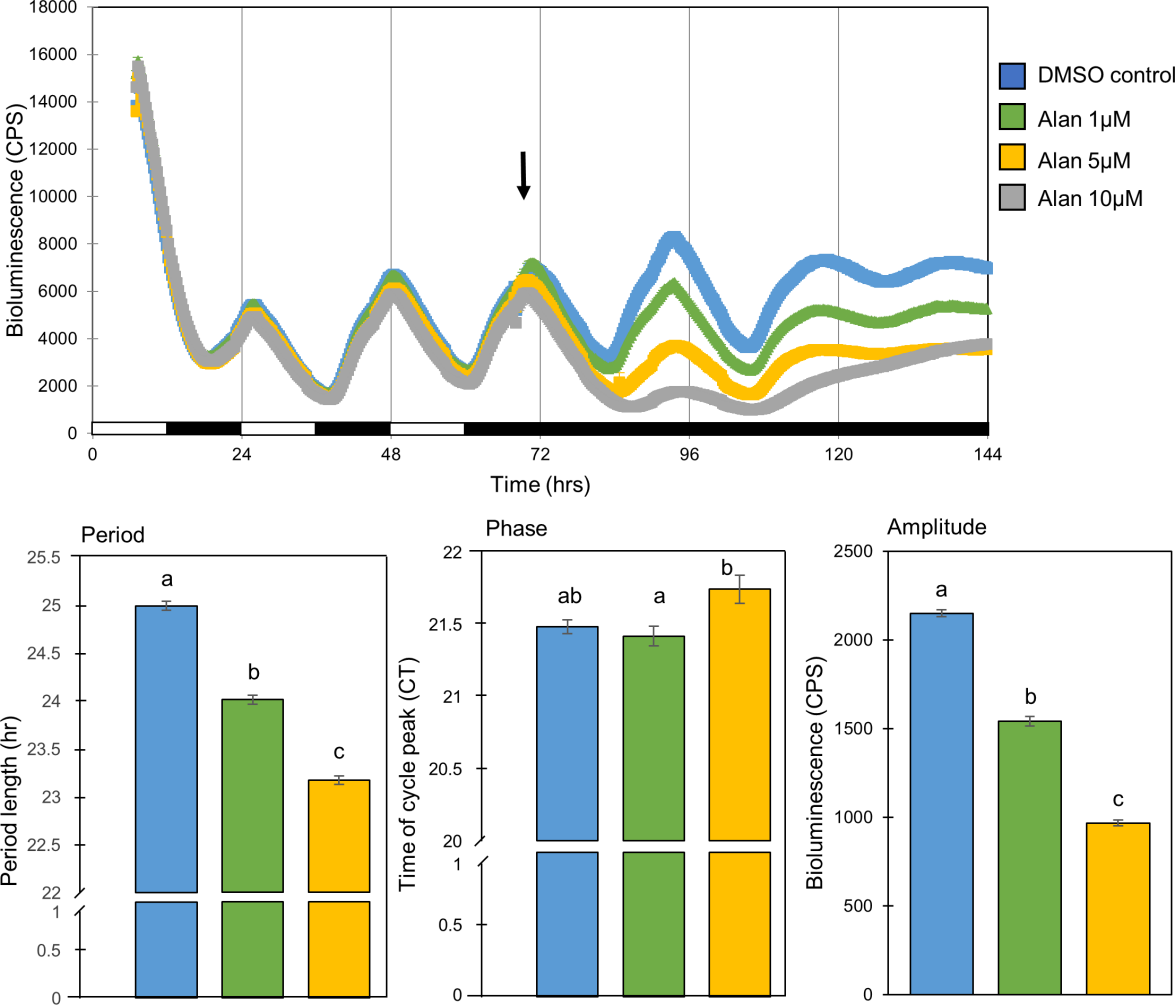
The molecular circadian oscillator in PAC-2 cells is significantly altered by TGF-β induction. Rhythmic *Per1b* promotor activity in the zebrafish PAC-2 cell line was significantly altered by the addition of the indirect TGF-β inducer Alantolactone to the culture media in a dose-dependent manner in comparison to DMSO-treated control (n = 8/group). *Upper panel*: bioluminescence is plotted on the y-axis and time (hours) on the x-axis. The horizontal bars represent the lighting conditions during bioluminescence measurements; white boxes represent light periods and black boxes represent dark periods. *Lower panel*: cells which were exposed to Alantolactone exhibit lower amplitude (*p<0*.*001*, one-way ANOVA), a phase delay (*p<0*.*001*, one-way ANOVA), and shorter periods of rhythms (*p<0*.*001*, one-way ANOVA). Different letters represent statistically different values within each parameter (*p<0*.*05*, Tukey’s test).

The addition Alantolactone to the culture media disrupted the clock controlled rhythmic activity of the *per1b* promotor (Fig 4). It led to a significant dose-dependent reduction in the amplitude of rhythmic *Per1b* promotor activity under DD (1542.31±27.76, 968.69±16.07 CPS for 1 and 5 μM Alantolactone respectively, compared to 2151.31±18.44 for the DMSO-treated, during the first DD cycle after exposure, *p<0*.*001*, one-way ANOVA, *p<0*.*001*, Tukey's post-hoc). The period of *Per1b* promotor activity was reduced by the activator (24.01±0.05, 23.17±0.05 hr for 1 and 5 μM, respectively, compared to 24.99±0.05 for the DMSO treated control, *p<0*.*001*, one-way ANOVA, *p<0*.*05*, Tukey's post-hoc). However, Alantolactone was not found to effect *per1b* activity phase (the time of the first peak after the cells were transferred to DD was at CT 21.42±0.07, 21.74±0.1 for 1 and 5 μM, respectively, compared to CT 21.48±0.05 for the DMSO-treated control, *p<0*.*001*, one-way ANOVA, *p<0*.*05*, Tukey's post-hoc). At the highest tested concentration (10μM) Alantolactone totally abolished the rhythm; amplitude, period and phase were therefore not calculated for the 10μM Alantolactone-treated cells.

#### 2.2 TGF-β inhibition leads to phase delay of *per1b* mRNA rhythms in zebrafish larvae

**Fig 5:**
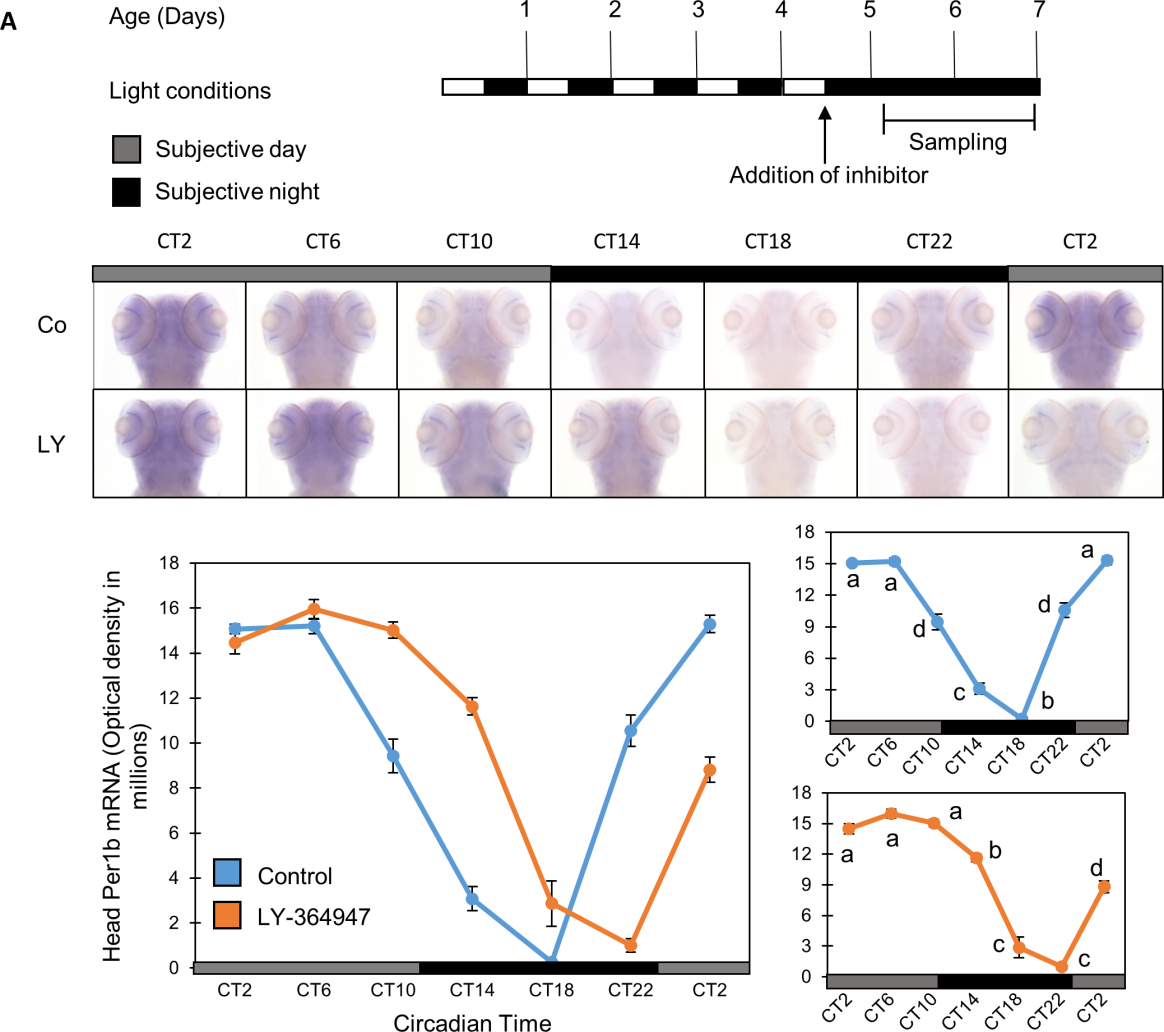
*Per1b* mRNA circadian expression pattern in zebrafish larvae is phase-shifted by TGF-β inhibition. Zebrafish larvae were treated with TGF-β inhibitor LY-364947 (20 μM), and the expression pattern of *Per1b* was evaluated by whole mount ISH. *Per1b* expression was detected throughout the head region and its circadian expression pattern was altered in the presence of the TGF-β inhibitor, exhibiting a phase delay of circadian expression in comparison to a control group (DMSO). *Per1b* mRNA expression was significantly affected by sampling time (*p<0*.*001*, two-way ANOVA), and by an interaction between treatment and sampling time (*p<0*.*001*, two-way ANOVA) (n = 15/group). (A) Schematic representation of the experimental design. The horizontal bars represent the light conditions before and during sampling; white boxes represent light and black boxes represent dark periods. *Bottom panel*: whole mount ISH signals for *Per1b* mRNA (dorsal views of the heads) of representative specimens. Grey bars represent subjective day and black bars represent subjective night. Circadian times are indicated for each sample. CT0 corresponds to “subjective lights on”, CT12 to “subjective lights-off”. (B) *Left*: Quantification of signal intensities in the heads of treated and control larvae. Values represent the mean ± SE optical densities of the head signals. *Right*: Different letters represent statistically different values within each treatment (*p<0*.*05*, one-way ANOVA, Tukey’s test). This experiment was repeated twice, resulting in similar outcomes. The represented results are of one experiment.

After demonstrating that pharmacological inhibition of TGF-β influences the circadian clock of zebrafish cell lines *in vitro*, we next evaluated the influence of this inhibition at the whole organism level by testing its effect on the clock-controlled rhythmic expression pattern of *Per1b* mRNA. Zebrafish larvae were kept under LD cycles for 5 days. Near the end of the light phase of the 5th day of development, approximately 30 min before the lights were turned on, the TGF-β inhibitor LY-364947 (20µM) or diluted DMSO alone (control) was added to the larvae water, and larvae were transferred to DD conditions. During the 6^th^ and 7^th^ days of development fish were collected at 4 hr intervals and *Per1b* mRNA levels were measured by whole mount ISH (n = 15/group). *Per1b* mRNA expression was significantly affected by sampling time (*p<0*.*001*, two-way ANOVA), and there was a significant interaction between treatment and sampling time (*p<0*.*001*, two-way ANOVA). Thus, the circadian expression pattern of *Per1b* mRNA was significantly altered in larvae exposed to the TGF-β inhibitor, demonstrating a phase delay of circadian expression in comparison to the control group (Fig 5). The observed phase delay is similar to the phase delay of *Per1b* promotor activity rhythms in PAC-2 cells upon exposure to the TGF-β inhibitor (Fig 3). These results indicate that TGF- β signaling influences the rhythmic transcription of a core clock gene in zebrafish larvae.

#### 2.3 TGF-β inhibition reversibly disrupts clock-controlled rhythmic locomotor activity in zebrafish larvae

**Fig 6:**
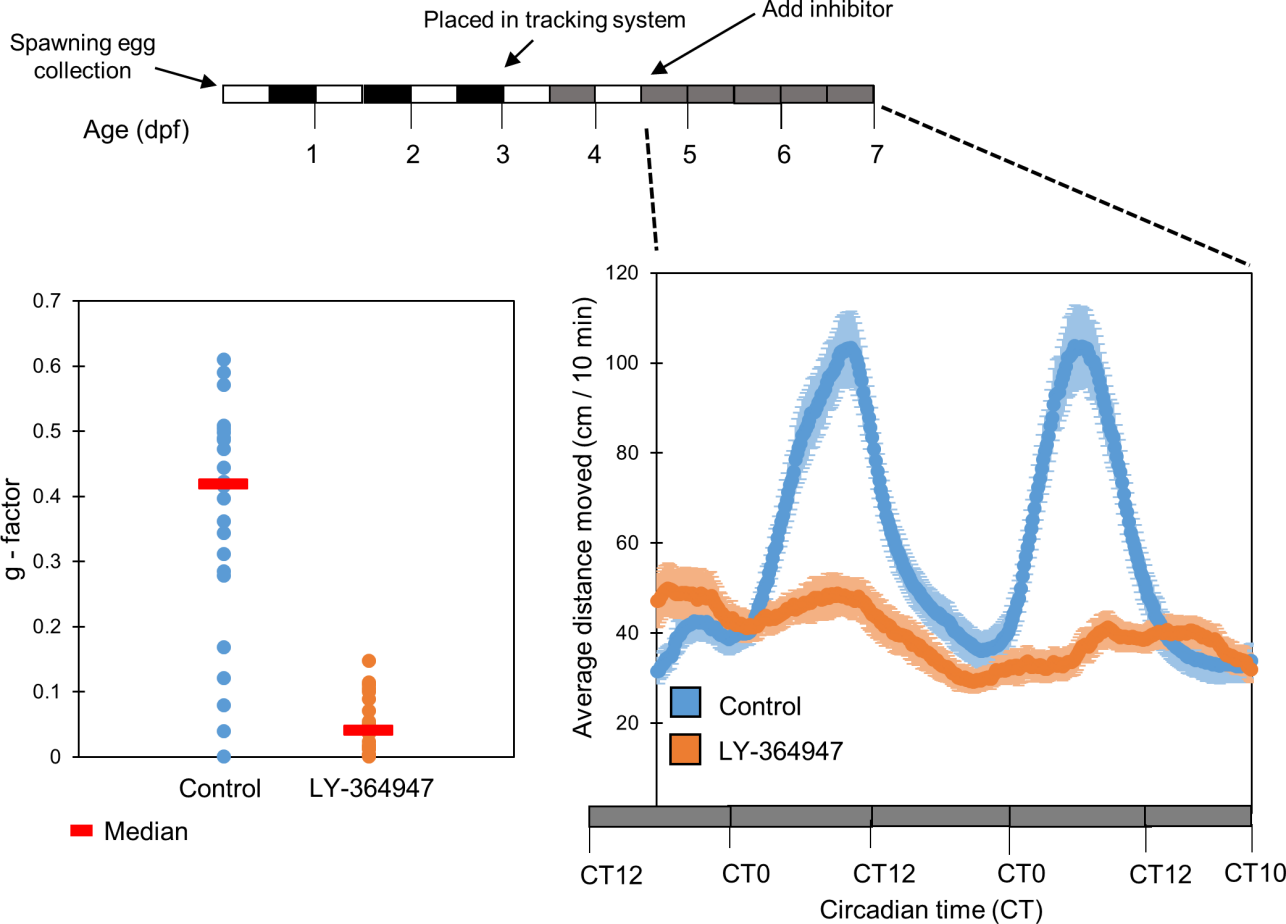
TGF-β inhibition abolishes clock-controlled rhythmic locomotor activity in zebrafish larvae. Clock-controlled rhythmic locomotor activity of zebrafish larvae under constant dim light was abolished after treatment with the TGF-β inhibitor LY-374947 (20µM) in comparison to a control group (DMSO). Embryos were raised under LD for 3 days, raised under LDim in the DanioVision chamber for 2 days, the inhibitor was then applied and locomotor activity (distance moved every 10 min) was monitored under constant Dim. The data is presented as a moving average (10 sliding points) for each group (n = 24/group). Larvae exhibited a significant reduction in the amplitude of rhythmic locomotor activity (*p<0*.*001*, *t-test*, bottom right panel). The horizontal bars represent the lighting conditions before and during the experiment. White boxes represent light, black boxes represent dark and grey boxes represent dim light (upper panel). TGF-β inhibitor-treated larvae exhibited significantly lower g-factor values (fitness to a circadian rhythm) in comparison to control larvae (*p<0*.*001*, Kolmogorov-Smirnov test), indicating that their locomotor activity is less circadian (bottom left panel). The median is represented for each group (red line).

Studies of the influence of TGF-β signaling on the circadian clock have been limited so far to its influence on the core molecular mechanism [19,20]. Therefore, we next aimed to test whether TGF-β signaling also influences a behavioral output of the clock, namely clock-controlled circadian rhythms of locomotor activity [4,32]. The influence of a TGF-β inhibitor on larval locomotor activity was tested following a previously described experimental protocol [33]. Larval clocks were entrained by exposure to 3 LD cycles and two 12 hr light:12 hr dim light cycles (LDim) and then transferred to constant dim light (DimDim). Locomotor activity was recorded under DimDim during the 6^th^-7^th^ days of development in the presence of the TGF- β inhibitor LY-364947 (20 µM) which was added to the larvae water during the 5^th^ day of development (n = 24/group). Circadian rhythms of locomotor activity were significantly affected in larvae treated with the TGF-β inhibitor in comparison with the DMSO treated control group (Fig 6; *p<0*.*001*, Kolmogorov-Smirnov test).

Inhibitor-treated larvae exhibited a significantly lower amplitude (1.52±0.48 and 4.15±0.84 cm/10 min for inhibitor-treated and control larvae respectively, *p<0*.*001*, *t*-test), similar to the decrease in amplitude of *per1b* promotor activity following TGF-β inhibition observed *in vitro*.

**Fig 7:**
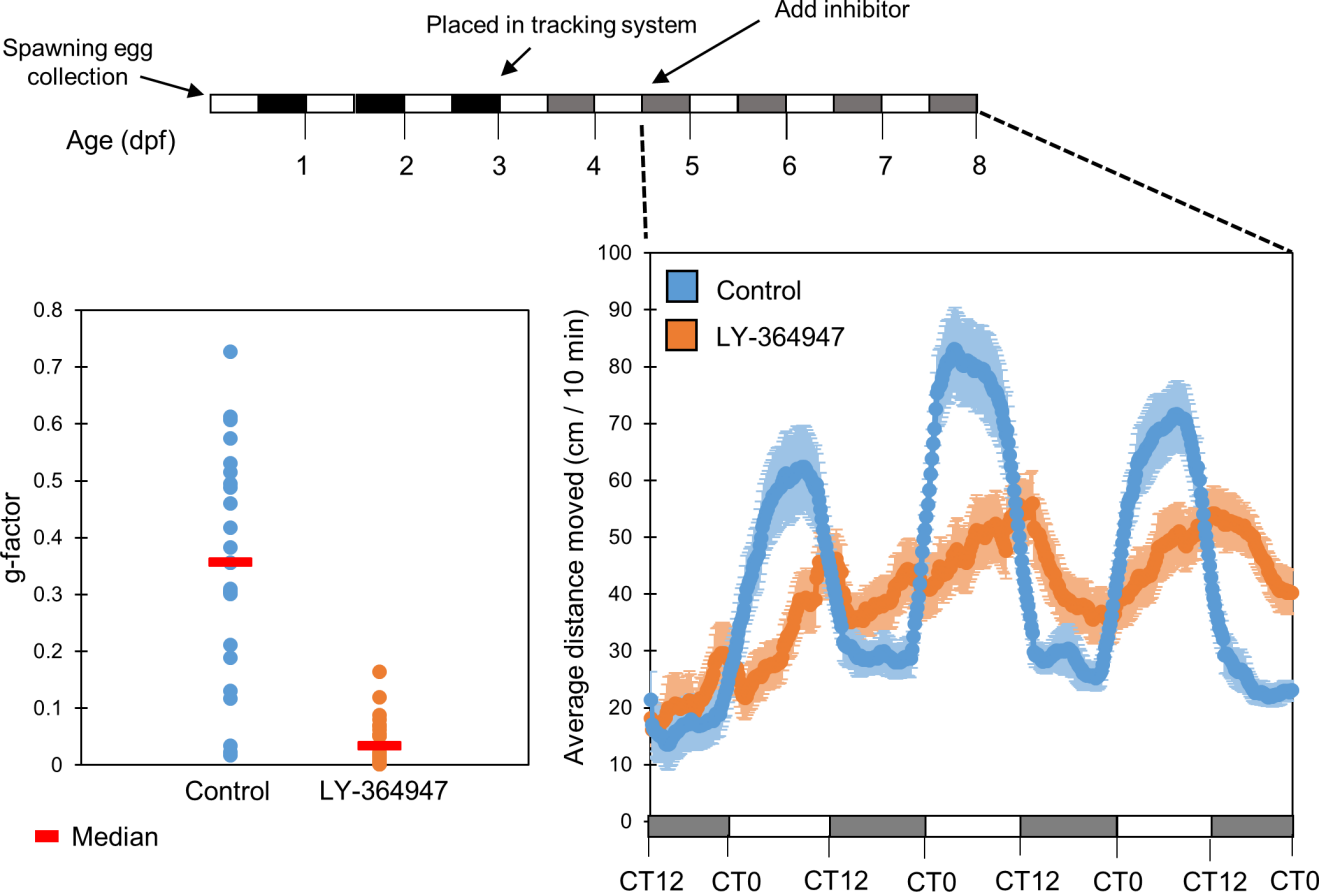
TGF-β inhibition disrupts circadian locomotor activity rhythms under light:Dim light cycles. Larval rhythmic locomotor activity under LDim was significantly disrupted (*p<0*.*05*, *t-*test), but not completely abolished, after treatment with the TGF-β inhibitor LY-374947 (20µM) in comparison with the DMSO control group. Embryos were raised under LD for 3 days, raised under LDim in the DanioVision chamber for 2 days, the inhibitor was added and locomotor activity (distance moved every 10 min) was monitored under LDim cycles. The data is presented as a moving average (10 sliding points) for each group (n = 24/group). The horizontal bars represent the lighting conditions before and during the experiment. White boxes represent light, black boxes represent dark and grey boxes represent dim light (upper panel). TGF-β inhibitor treated larvae exhibited significantly lower g-factor values in comparison with control larvae (*p<0*.*001*, Kolmogorov-Smirnov test), indicating that their locomotor activity is significantly less circadian LDim cycles. The median is represented for each group (red line). This experiment was repeated twice, resulting in similar outcomes. The represented results are of one experiment.

Light exposure has been extensively documented to have an acute effect on the locomotor activity of zebrafish larvae, independently of regulation by the endogenous circadian clock [27]. Therefore, we next aimed to determine whether this “masking” effect of light could overcome the effect of the TGF-β inhibitor, and restore or prevent disruption in rhythmic locomotor activity of the larvae. Larvae were entrained to 3 LD and 2 LDim cycles and locomotor activity was monitored on the 6^th^-8^th^ day of development under LDim cycles in the presence or absence of the inhibitor (n = 24/group). Circadian rhythms of locomotor activity were significantly altered in larvae treated with TGF-β inhibitor in comparison to the DMSO treated control group (*p<0*.*001*, Kolmogorov-Smirnov test; Fig 7). Inhibitor-treated larvae exhibited a significantly longer period of rhythmic locomotor activity (25.86±0.75 and 23.19+1.34 hr for treated and control larvae, respectively, *p<0*.*05*, *t-*test). Consequently, inhibitor-treated larvae displayed a delayed phase (peaking at CT11±4.4 and CT5±5.22 hr for inhibitor-treated and control larvae, respectively, *p<0*.*001*, *t-*test), reminiscent of the period lengthening and phase delay observed in the activity of *Per1b* promotor activity *in vitro* (Fig 3), and the phase delay in *per1b* mRNA expression *in vivo* (Fig 5). Treated larvae also exhibited a lower amplitude rhythm (2.49±1.02 and 3.98±1.16 cm/10 min for inhibitor treated and controlled larvae, respectively, *p<0*.*05*, *t-*test), reminiscent of the decrease in the amplitude of *Per1b* promotor activity *in vitro*. The alteration of locomotor activity circadian rhythms, even under LDim cycles, further reinforces the importance of TGF-β signaling for the function of the circadian system.

**Fig 8:**
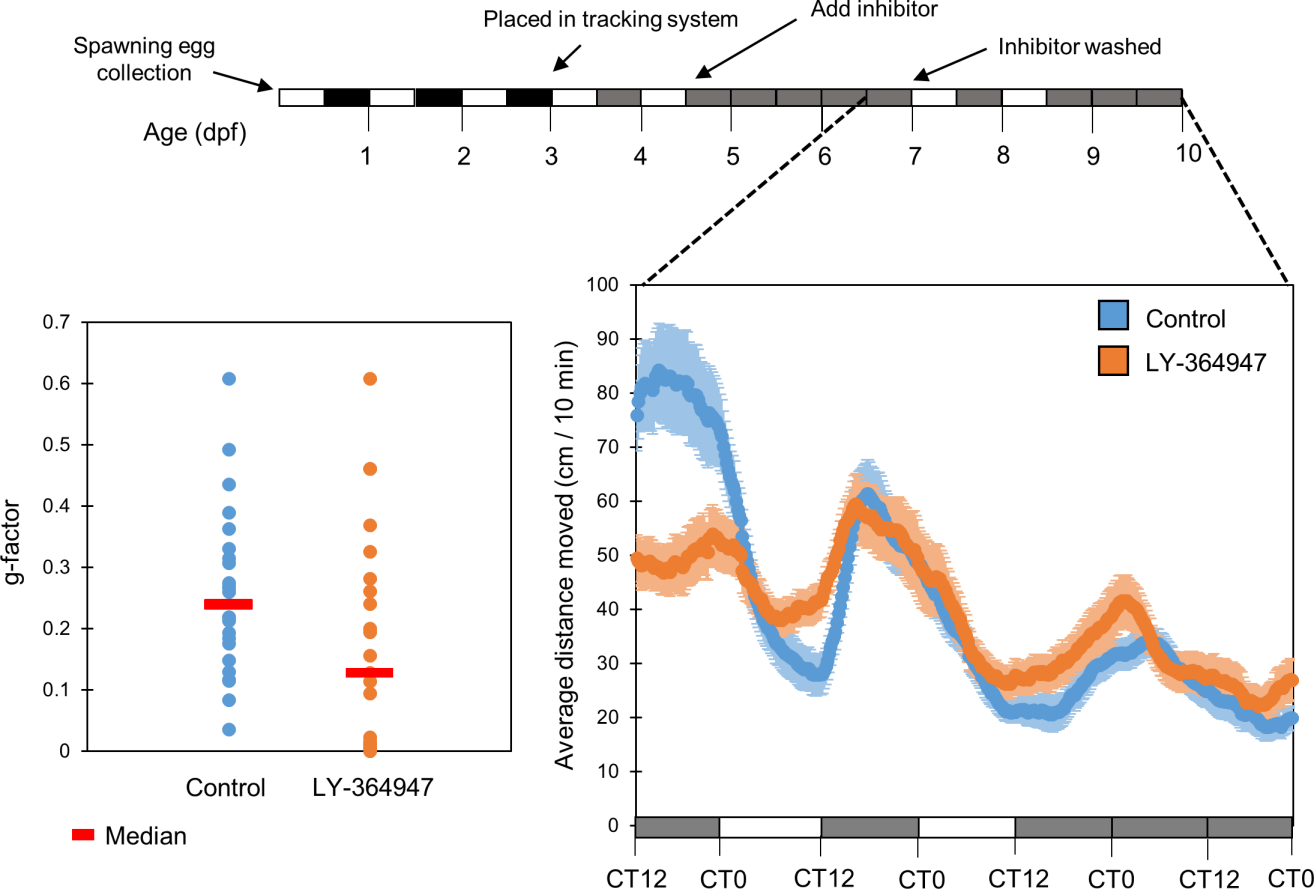
The effect of TGF-β inhibition on clock-controlled rhythmic locomotor activity in zebrafish larvae is reversible. Embryos were raised under LD for 3 days, raised under LDim in the DanioVision chamber for 2 days, the inhibitor (20μM LY-374947) was applied for an additional 2 DimDim cycles. After washing off the inhibitor, the larvae were entrained by 2 LDim cycles and locomotor activity (distance moved every 10 min) was monitored under constant Dim. Following removal of the TGF-β inhibitor, normal circadian rhythmicity of locomotor activity in treated larvae was recovered. A day following inhibitor wash out there were no significant differences in the g-factor distribution between DMSO and inhibitor treated larvae (*p = 0*.*12*, Kolmogrov-Smirnov test), as well as no significant differences in amplitude (*p = 0*.*32*, *t-*test), phase (*p = 0*.*73*, *t-*test) or period length (*p = 0*.*58*, *t-*test) (bottom left panel). The data is presented as a moving average (10 sliding points) for each group (n = 23/group). The median is represented for each group (red line). This experiment was repeated twice, resulting in similar outcomes. The represented results are of one experiment.

Given the striking effect of pharmacological TGF-β inhibition on larval circadian locomotor activity, and to rule out the possibility of an irreversible toxic effect, we examined whether this effect could be reversed. In order to address this issue, we performed an inhibitor “wash-out” experiment: larvae were kept under LD cycles during the first 5 days of development, and then placed in DD during the 6^th^-7^th^ days of development in the presence of TGF-β inhibitor LY-364947 (20µM). On the morning of the 8^th^ day of development, the inhibitor was removed by washing. Larvae were re-entrained by two LDim cycles, and then kept under DimDim conditions for an additional 24 hours, during which their locomotor activity was recorded (n = 23/group). 24 hours after removal of the inhibitor, normal circadian rhythmicity of locomotor activity in inhibitor-treated larvae was completely recovered (Fig 8). 24 hours following inhibitor washout there were no significant differences in the g-factor distribution between control and inhibitor-treated larvae (*p = 0*.*12*, Kolmogorov-Smirnov test), as well as no significant difference in amplitude (2.37±0.31 and 2.8±0.63 cm/10 min for inhibitor-treated and control larvae, respectively, *p = 0*.*32*, *t-*test) period length (24.77±0.26 and 25.19±0.52 hr for treated and control larvae, respectively, *p = 0*.*58*, *t-*test) or phase (CT7.5±1.07 and CT8±0.28 hr for treated and control larvae respectively, *p = 0*.*73*, *t-*test). This indicates that the effect of pharmacological TGF-β inhibition on circadian rhythms of locomotor activity is reversible.

**Fig 9:**
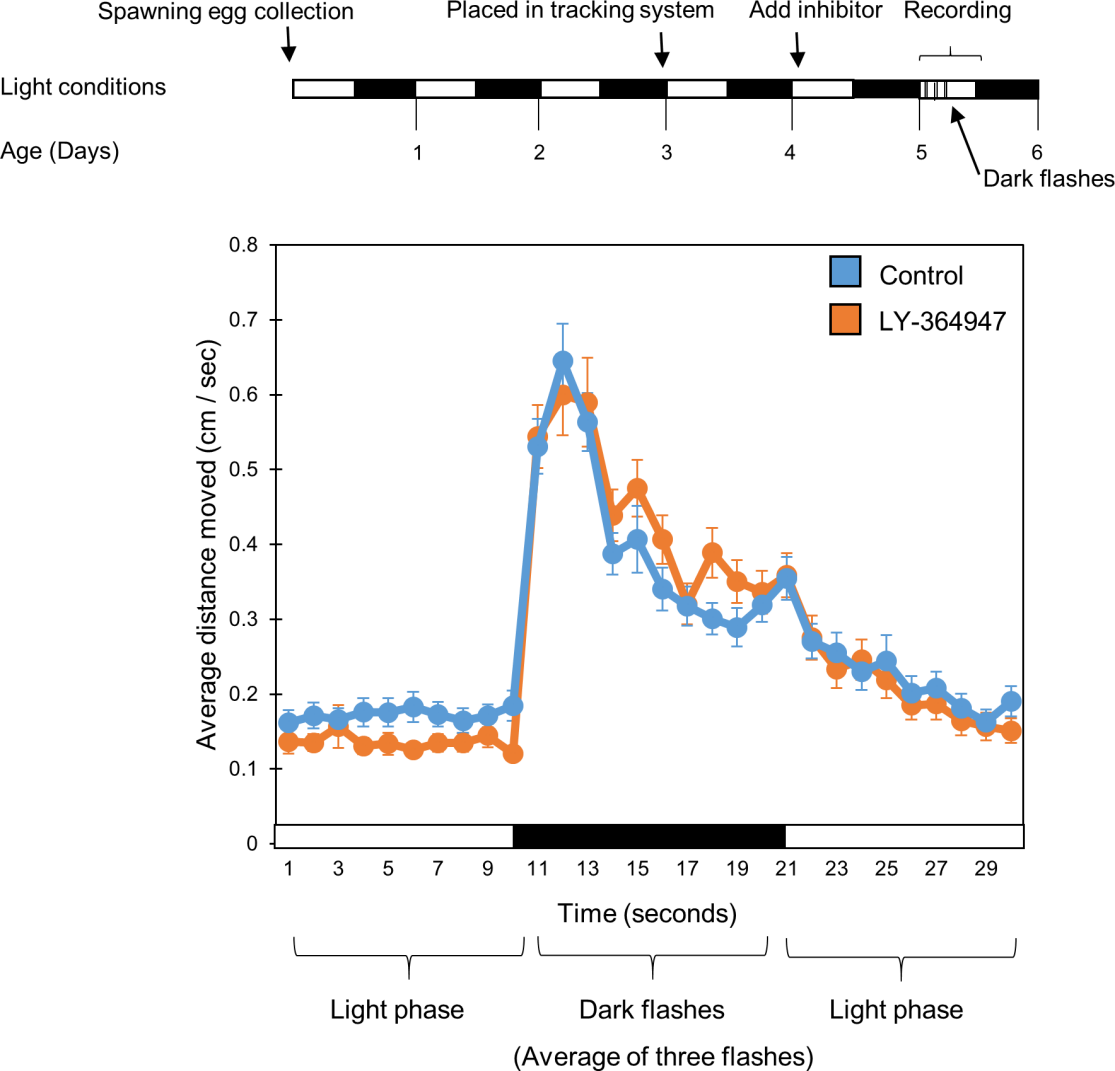
Locomotor activity levels in response to dark flashes is not effected by TGF-β inhibition. Larvae were kept under LD cycles. On day 5 the inhibitor, or DMSO as control, was added on day 6. Larvae were subjected to 3 dark flashes of 10 seconds each, which are known to induce startle response, with 15 minutes intervals of light between flashes, and their activity was recorded (upper panel). No statistical difference was observed between the activity of control (DMSO) and the TGF-β inhibitor (LY-374947, 20μM) treated groups during the dark flashes (*p = 0*.*28*, *t*-test), indicating that TGF-β inhibition does not impair larval mobility (lower panel). Each line represents the average of three succeeding trials, which measured the average movement per second of each group of larvae, recorded from 10 second before the flash, during the flash, and 10 second after the flash. Black and white horizontal boxes represent the light phase and dark flashes, respectively. This experiment was repeated twice, resulting in similar outcomes. The represented results are of one experiment.

To rule out the possibility that LY-364947 simply impairs larval mobility, we performed an additional assay for the behavioral response to light-to-dark transitions The behavioral response of the larvae to a sudden light transition is thought to be a locomotor behavior not regulated by the circadian clock [34], and therefore serves as a valuable parameter to test whether the TGF-β inhibitor affects larvae’s mobility. During the early light phase of the 6^th^ day of development, larvae were subjected to 3 dark flashes of 10 seconds each, with 15 minutes of light interval between flashes, in the presence of the TGF-β inhibitor LY-364947 (20µM). Locomotor activity was recorded before, during and after the dark flashes. No statistical difference was observed between the response of inhibitor-treated and control DMSO treated larvae (n = 24/group) to dark flashes (Fig 9; *p = 0*.*28*, *t*-test), indicating that LY-364947 does not impair larval mobility.

## Discussion

Previous studies have implied the presence of a functional link between the circadian clock and TGF-β signaling [19–22,26,35], however, this connection has not been thoroughly characterized. In the present study, by data mining of transcriptome data, and the study of zebrafish cell-lines *in vitro* as well as by *in vivo* analysis, we demonstrate interactions between the circadian clock and the TGF-β signaling pathway at the molecular and behavioral levels. Furthermore, we show that TGF-β is necessary for normal circadian clock function.

Data mining of microarray and RNAseq experiments reinforces previous studies indicating that *Smad3* expression exhibits circadian oscillations [21,22], and reveals that these oscillations occur both in whole zebrafish larvae and adult zebrafish brains, with a similar period, peaking at the beginning of the subjective day. Furthermore, data mining reveals that the mRNA of two additional TGF-β related gene, *Smad7* and *Tgif1*, also exhibit clock-controlled circadian rhythms in the whole zebrafish embryo and in adult zebrafish pineal glands (but not in the adult zebrafish brain), with a similar period, peaking at the middle of the dark period. The E-box and RORE promoter enhancer elements have been demonstrated to direct circadian rhythms of gene expression in CCGs by the core clock transcription factor components Clock:Bmal and RevErb/ROR respectively [2,3]. Interestingly, in zebrafish the phase of rhythmic expression directed by these two enhancer elements differs by around 12 hours, with E-box (Clock:Bmal) driven expression peaking in the early light period while RORE (RevErb /ROR) driven expression peaks during the early night [2]. It is therefore tempting to speculate that the rhythms of TGF-β related gene expression may reflect core clock regulation via both types of enhancer element.

Whole mount ISH results validate and reinforce transcriptome analysis data indicating that *Smad3a* mRNA exhibits a widespread circadian expression pattern in the whole head area of zebrafish larvae. Previous results have demonstrated that *Smad3a* oscillates both in central circadian clock organs, namely the mouse SCN and zebrafish pineal [22], in various cell-lines and *in vivo* in the mouse liver [21]. Earlier transcriptome experiments and the current experimental results strengthen the notion that *Smad3a* exhibits a rhythmic circadian expression pattern in multiple peripheral tissues, which is also affected by the photic regime. Although previous studies in mammals have suggested that the expression of TGF-β itself is regulated by the circadian clock [22,35], our own zebrafish transcriptome analysis failed to detect a circadian expression pattern for either *Tgfb1*, *Tgfb2*, or *Tgfb3*. It is therefore possible that this extra layer of regulation of TGF-β signaling by the circadian clock in mammals may have evolved following the divergence of the teleost lineage from other vertebrates, and therefore appears in mammals but not in zebrafish. Due to the lack of appropriate antibodies, we examined the circadian profile of zebrafish TGF-β signaling elements only at the mRNA level, and not at the protein or active protein levels. Therefore, it remains to be tested precisely how the rhythms in mRNA influence protein levels of Smad3, phosphorylated Smad3 and TGF-β in zebrafish. However, given that cycling protein levels for TGF-β signaling pathway elements have been described in the mammalian SCN [22], it seems likely that a comparable protein rhythmicity also exists in zebrafish.

We show that pharmacological inhibition of TGF-β causes period lengthening, a consequent phase delay and a decrease in amplitude of rhythmic expression of the core clock gene *Per1b* in PAC-2 cells, and a phase delay in the rhythmic expression of *Per1b* mRNA *in vivo*. Since Per1b plays a key role in the molecular mechanism of the circadian clock [1], a change in *Per1b* promotor activity and mRNA expression reflects alterations in the expression of other clock genes and indicates a general shift in the molecular mechanism of the circadian clock.

Interestingly, pharmacological indirect activation of TGF-β signaling using Alantolactone also disrupted the rhythmic expression of the core clock gene *Per1b* in PAC-2 cells, causing a decrease in amplitude and a period shortening. Such results might indicate a significant disruption in TGF-β signaling, either decrease or increase, disrupts the activity of the molecular circadian clock. It is important to note some of the effects observed in the indirect activation of TGF-β experiments might be due to unknown effects of the compound Alantolactone on other cellular pathways [31]. Importantly, it should be noted that previous evidence suggest Alantolactone does not have any largely visible effects on non-cancerous cells [31].

The observed alternations in the period and phase of *Per1b* rhythmic expression as a result of pharmacological inhibition or indirect activation of TGF-β are largely consistent with previous evidence from Kon et al [19], showing that intraperitoneal injection of TGF-β towards the end of the night caused a 3 hour advance in rhythmic *Per1* expression in the kidney and adrenal gland. In the current study only TGF-B inhibition seemed to cause a delay in the phase of *Per1b* rhythmic expression, while indirect activation did not affect the phase, but caused period shortening, which is usually associated with phase advance. These difference might be since our manipulation, namely the addition of a TGF-β inhibitor or indirect activator to the zebrafish water, is inherently different from the manipulation performed by Kon et al., in the length of the treatment (chronic vs. acute).

Pharmacological inhibition of TGF-β affected not only the molecular circadian clock, but also clock-controlled behavior, as TGF-β inhibition disrupted the clock-controlled rhythms of locomotor activity of larvae. This effect was evident under constant lighting conditions as well as under LDim cycles, which failed to mask the effect of the inhibitor. General locomotor ability was not affected, as indicated by the response to dark flashes. Furthermore, the period lengthening and phase delay in the rhythmic activity of inhibitor-treated larvae closely resemble the period lengthening and phase delay in the activity of the *Per1b* promotor *in vitro*. Therefore, we conclude that the effect of TGF-β inhibition on clock-controlled behavior can be explained by its effect on components of the molecular circadian clock. In this regard, it will be interesting to test whether TGF-β inhibition influences other circadian controlled behaviors, such as temporal feeding patterns [36]. In the future, a complementary approach of computational modelling may be valuable to further elucidate the mechanism linking the circadian clock and TGF-β signaling. Computational modelling of regulatory networks has proven highly valuable in analyzing and understanding system level phenomena [37–39]. Network models of a discrete nature are one type of computational model that usually do not require detailed quantitative biological data. Software tools that implement computational models can be used to simulate network behavior and allow extensive *in silico* exploration of the network performance under numerous simulated conditions. Such a tool called BioNSi (Biological Network Simulator) was recently used to simulate the molecular mechanism of the vertebrate circadian clock, including its bidirectional interactions with the TGF-β signaling pathway [40]. Such a software tool will also be extremely useful to study the interactions between the molecular circadian clock and the TGF-β signaling in zebrafish, in order to identify plausible underlying mechanisms and make new predictions that can then be tested experimentally.

The interactions between the circadian clock and TGF-β signaling are especially intriguing considering their possible influence on outputs of both systems. The circadian expression pattern of *Smad3* can control the timing of Smad2/3:Smad4 dependent transcription, and thus cause circadian oscillations in Smad-controlled genes. On the other hand, the influence of Smad2/3:Smad4 on *Per1* and perhaps on additional clock genes affects the circadian molecular oscillator, in turn potentially influencing the expression of CCGs. These bi-directional interactions may lead to many interesting effects. For example, outputs that are usually known to be regulated by the circadian clock may be found to be also regulated by TGF-β, and vice versa. In addition, these two systems may also exhibit shared complex effects, such as opposite, additive or synergistic effects. This is especially interesting since shared outputs of TGF-β signaling and the circadian clock include the cell cycle and apoptosis, and common outcomes upon disruption of these two interconnected systems include tumorigenesis and tumor progression.

## Materials and methods

### 1. Transcriptome data mining

Transcriptome data mining was performed on three previous transcriptome analysis experiments which were performed on whole zebrafish larvae [23], adult zebrafish brains [24], and adult zebrafish pineal glands [26].

### 2. Fish maintenance

Adult zebrafish were raised in a recirculation water system at the zebrafish facility of Tel Aviv University under 12hr light:12hr dark (LD) cycles at 28°C and fed twice each day. To generate embryos, male and female zebrafish were paired in the evening, and spawning occurred the next day within one hour after lights on. Embryos were placed in 10 cm petri dishes with egg water containing methylene blue (0.3 p.p.m) and raised under LD cycles at 28°C. For whole mount ISH, pigmentation was prevented by adding phenylthiourea (PTU) to the embryos water during the first two days of development. For locomotor activity analysis, embryos were transferred into 48 plates (one larva per well) during the fourth day of development and placed into the DanioVision observation chamber (Noldus Information Technology, the Netherlands). All procedures were approved by the Tel Aviv University Animal Care Committee and conducted in accordance with the Council for Experiments on Animal Subjects, Ministry of Health.

### 3. Whole mount ISH

Samples were collected at 4 hr intervals throughout the 24 hr cycle during the 6^th^ day of development, fixed for 24 hours in 4% paraformaldehyde and stored in 100% methanol at -20°C. Exposure to the TGF-β inhibitor LY-364947 at a concentration of 20μM began on the evening of the 5^th^ day of development. Transcripts of *Smad3a*, *Smad3b* and *Per1b* mRNA were detected by whole mount ISH using digoxygenin-labelled antisense ribo-probes (DIG RNA labelling kit, Roche Diagnostics Ltd, Basel, Switzerland). Probes were produced as previously described and whole mount ISH analyses were carried out according to an established protocol [41]. Whole mount ISH signals in the larva head, expressed as optical density, were quantified using ImageJ software (National Institute of Health, Bethesda, MD, USA). The larva head area was chosen because of the higher expression of the studied genes in this region as compared to the trunk. Differences in signal intensities between treatments and sampling times were determined by two-way ANOVA. Specific comparison within each treatment were performed using one-way ANOVA followed by Tukey’s post-hoc test. Results are written as mean optical density ± standard error.

### 4. TGF-β inhibitors and indirect activator

Pharmacological inhibition of TGF-β signaling was carried out using a selective ATP-competitive inhibitor of TGF-β type-1 activin receptor-like kinase (ALK-5), LY-364947 (L6293, Sigma, MO), or a selective inhibitor of both ALK-4 and ALK-5, SB-431542 (S4317, Sigma, MO). Both inhibitors were previously demonstrated to inhibit TGF-β-Smad3 mediated signaling in zebrafish larvae [30]. For *in vitro* experiments, the inhibitors were dissolved in DMSO and added at working concentrations of 1, 5, 10 and 20 μM to the cell culture medium, 30 minutes before lights on. For *in vivo* experiments, LY-364947 was dissolved in DMSO and was added to the larvae water during the evening of the 5^th^ day of development, before lights off, at a final concentration of 20μM.

The LY-364947 concentrations used in current experiments are higher than reported IC50 values in cell-free binding assays, which are about 0.04–0.1 μM for the target of interest, TGFβRI [42,43]. Similar IC50 values have also been reported in several previous cell-line based experiments [43–45]. However, multiple studies have used much higher concentrations of LY-364947 in cell-cultures, ranging from 5μM [46] and 10μM [47] to as high as 40μM [48]. LY-364947 has not been previously used with zebrafish PAC-2 cells, but has been widely used with zebrafish embryos, consistently at working concentrations of 30–100μM [30,49–53]. Thus, concentrations that are higher than the IC50 have been routinely used to disrupt TGF- β signaling in cell and animal models. Accordingly, concentrations of 1–20 μM were used in the current study, consistent with the most commonly used concentrations in previous studies using the zebrafish model.

Pharmacological induction of TGF-β signaling was carried out using Alantolactone (SML0415, Sigma, MO), a sesquiterpene lactone which disrupts the Cripto-1/ActRII complexes, resulting in an indirect induction of activin/Smad3 signaling [31].

### 5. Cell cultures, constructs and real-time bioluminescence assays

The zebrafish PAC-2 cell line stably expressing *per1b*::*luc* [54] were cultured and entrained to LD cycles as described elsewhere [55,56]. 70 hours after entrainment, stably transfected cells were exposed to various concentrations of the TGF-β inhibitors LY-36494 or SB-431542, or the TGF-β indirect TGF- β inducer, Alantolacton. Control groups were treated with DMSO. Real-time bioluminescence assays were performed and analyzed as described previously [55,56], using an EnVision multilabel counter (Perkin Elmer).

The periods of luciferase rhythms while the cells were in DD conditions were computed by the Lomb-Scargle periodogram (α = 0.05) with Actogram software [57], and statistical differences between treated and control cells were determined by one-way ANOVA, followed by Tukey’s post-hoc test. Amplitude values were calculated as the difference between the peak during the first constant dark (DD) cycle after exposure and the following trough, divided by 2, and the statistical differences between treated and control larvae were determined by one-way ANOVA, followed by Tukey’s post-hoc test. Phase values were calculated as the CT in which luciferase activity reached its peak during the first DD cycle after exposure has occurred, and the statistical differences between treated and control larvae were determined by one-way ANOVA, followed by Tukey’s post-hoc test.

### 6. Locomotor activity monitoring of zebrafish larvae

For locomotor activity monitoring, Larvae were kept under LD conditions for three days as previously described [26], and on the 4^th^ day of development they were transferred into 48-well plates (one larva/well) and placed into a DanioVision observation chamber. The inhibitor, or DMSO, were added to the water near the end of the light phase of the 5^th^ day of development, approximately 30 minutes before lights-off, and larvae were then exposed to 12 hr light (3,400 lux): 12 hr dim light (40 lux) (LDim) cycles for 3 days, or to constant dim light, a condition in which larvae exhibit high amplitude clock-controlled rhythmic locomotor activity [58]. Live video tracking and analysis was conducted using the Ethovision 8.0 software (Noldus Information Technology). Activity was measured at 6–7 days post fertilization under DimDim or 6–8 days post fertilization under LDim, as the distance moved by a larva in 10 min time bins. The data is presented as a moving average (10 sliding points) for each group (n = 24/group).

For the "wash out" experiment, in which inhibitor was administered and then removed, larvae were kept under LD cycles, the inhibitor was added to the larvae water during the 5^th^ day of development. Starting on the 6^th^ day of development larvae were kept under DimDim for 60 hours. On the morning of the 8^th^ day of development the inhibitor was removed by washing, replaced with fresh water, and larvae were transferred into a 48 plate and placed into the DanioVision observation chamber. The larvae were re-entrained for 2 LDim cycles, and then kept under constant conditions (DimDim) for 24 hours, while locomotor activity (total distance moved by one larva during a 10 min time window) was measured using the Ethovision 8.0 software. The data is presented as a moving average (10 sliding points) for each group (n = 24/group).

Fourier analysis was used to test differences in rhythmic locomotor activity using a previously described procedure [26,28,33,58]. The time-dependent signal was converted into a frequency dependent signal using the Fast Fourier Transform (FFT). The extent to which the original signal of each larva is circadian was quantified by the ratio (‘g-factor’) of the power of the frequency that corresponds to the 24 hr period to the sum of powers of all frequencies. The higher the g-factor, the higher is the confidence that the larvae exhibit circadian locomotor activity. Differences in the g-factor distributions between the control and TGF-β inhibitor treated groups were determined by the Kolmogorov-Smirnov test. The periods of locomotor activity rhythms were computed by the Lomb-Scargle periodogram (α = 0.05) with Actogram software [57], and statistical differences between inhibitor treated and control larvae were determined by t-test. Amplitude values were calculated as the difference between the second recorded peak in activity and the preceding trough, divided by 2, and the statistical differences between inhibitor treated and control larvae were determined by t-test. Phase values were calculated as the difference between the CT of the second recorded peak of activity, and the statistical differences between inhibitor treated and control larvae were determined by one-way ANOVA.

For the “dark flash stimuli” experiment, used to observe larva mobility, larvae were placed in DanioVision during the 5^th^ day of development and exposed to one LD cycle. During early light phase on the 6^th^ day of development, the fish were subjected to 3 dark flashes of 10 seconds each, with 15 minutes of light interval between flashes. The data represents the average of three successive trials, which measured the average movement per second of each larvae, recorded 10 seconds before the flash, during the flash and 10 seconds after the dark flash.

## Supporting information

S1 Fig *Smad3b* mRNA does not show circadian expression in zebrafish larvae heads. *Smad3b* mRNA does not exhibit a circadian expression pattern in zebrafish larvae heads or other tissues. (A) Top panel: schematic representation of the experimental design. The horizontal bars represent the lighting conditions before and during sampling; white boxes represent light and black boxes represent dark periods. Bottom panel: Whole-mount ISH signals for *Smad3b* mRNA (dorsal views of the heads) of representative specimens raised under LD cycles until and during the sampling (LD group), or raised in DD during the sampling. Circadian times are indicated for each sample. CT0 corresponds to "subjective lights on", CT12 to "subjective lights-off". White bars represent light phases and black bars represent dark phases. (B) Quantification of signal intensities in the head of LD and DD larvae (n = 15/group). Values represent the mean ± SE optical densities of the head signals. White bars represent subjective day and black bars represent subjective night.
(TIF)

S2 Fig The molecular circadian oscillator in PAC-2 cells is significantly altered by TGF-β inhibition by SB-505124. Rhythmic *Per1b* promotor activity in the zebrafish PAC-2 cell line was significantly altered by the addition of the TGF-β inhibitor SB-505124 in a dose-dependent manner in comparison to DMSO treated control (n = 8/group). *Upper panel*: bioluminescence is plotted on the y-axis and time (hours) on the x-axis. The horizontal bars represent the lighting conditions before and during sampling; white boxes represent light periods and black boxes represent dark periods. *Lower panel*: effects of inhibition on length, phase, and amplitude of *Per1b* promotor activity. Different letters represent statistically different values within each parameter (*p<0*.*05*, one-way ANOVA, Tukey’s test). Treatment led to a significant lengthening of the period of *Per1b* promotor activity (23.76±0, 24.2±05, 24.2±0.05, 25.24±0.16 hr for 1, 5, 10 and 20 μM, respectively, compared to 24.35±0.12 for the DMSO-treated), and reduction in the amplitude (1313.75.25±9.37, 1128±24.02, 903±20.26, 594.19±23.29 CPS for 1, 5, 10 and 20 μM, respectively, compared to 2136.25±57.29 for the DMSO-treated control), but not to a significant phase delay (the time of the first peak after the cells were transferred to DD was at CT 3.74±0.12, 4.025±0.05, 3.92±1.5, 4.17±0.19 hr for 1, 5, 10 and 20 μM, respectively, compared to 3.85±0.07 for the DMSO-treated control).
(TIF)
