# Supplementary figures and images for: Interactions between the circadian clock and TGF-β signaling pathway in zebrafish
Source: PLoS One. 2018 Jun 25;13(6):e0199777. doi: 10.1371/journal.pone.0199777 (PMC6016920; doi:10.1371/journal.pone.0199777)

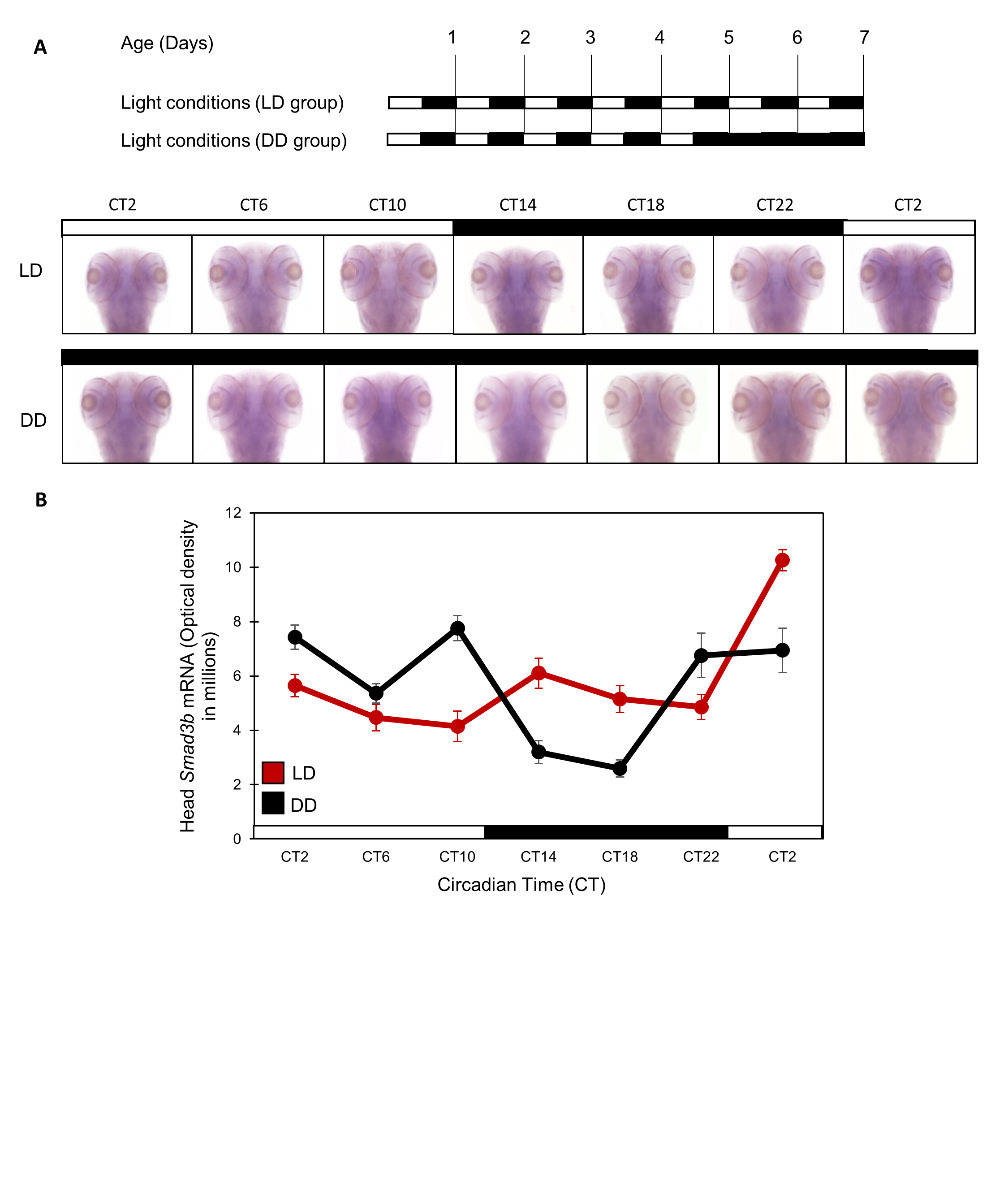

Supplement: S1 Fig — Smad3b mRNA does not exhibit a circadian expression pattern in zebrafish larvae heads or other tissues. (A) Top panel: schematic representation of the experimental design. The horizontal bars represent the lighting conditions before and during sampling; white boxes represent light and black boxes represent dark periods. Bottom panel: Whole-mount ISH signals for Smad3b mRNA (dorsal views of the heads) of representative specimens raised under LD cycles until and during the sampling (LD group), or raised in DD during the sampling. Circadian times are indicated for each sample. CT0 corresponds to "subjective lights on", CT12 to "subjective lights-off". White bars represent light phases and black bars represent dark phases. (B) Quantification of signal intensities in the head of LD and DD larvae (n = 15/group). Values represent the mean ± SE optical densities of the head signals. White bars represent subjective day and black bars represent subjective night. (TIF) [file pone.0199777.s001.TIF]

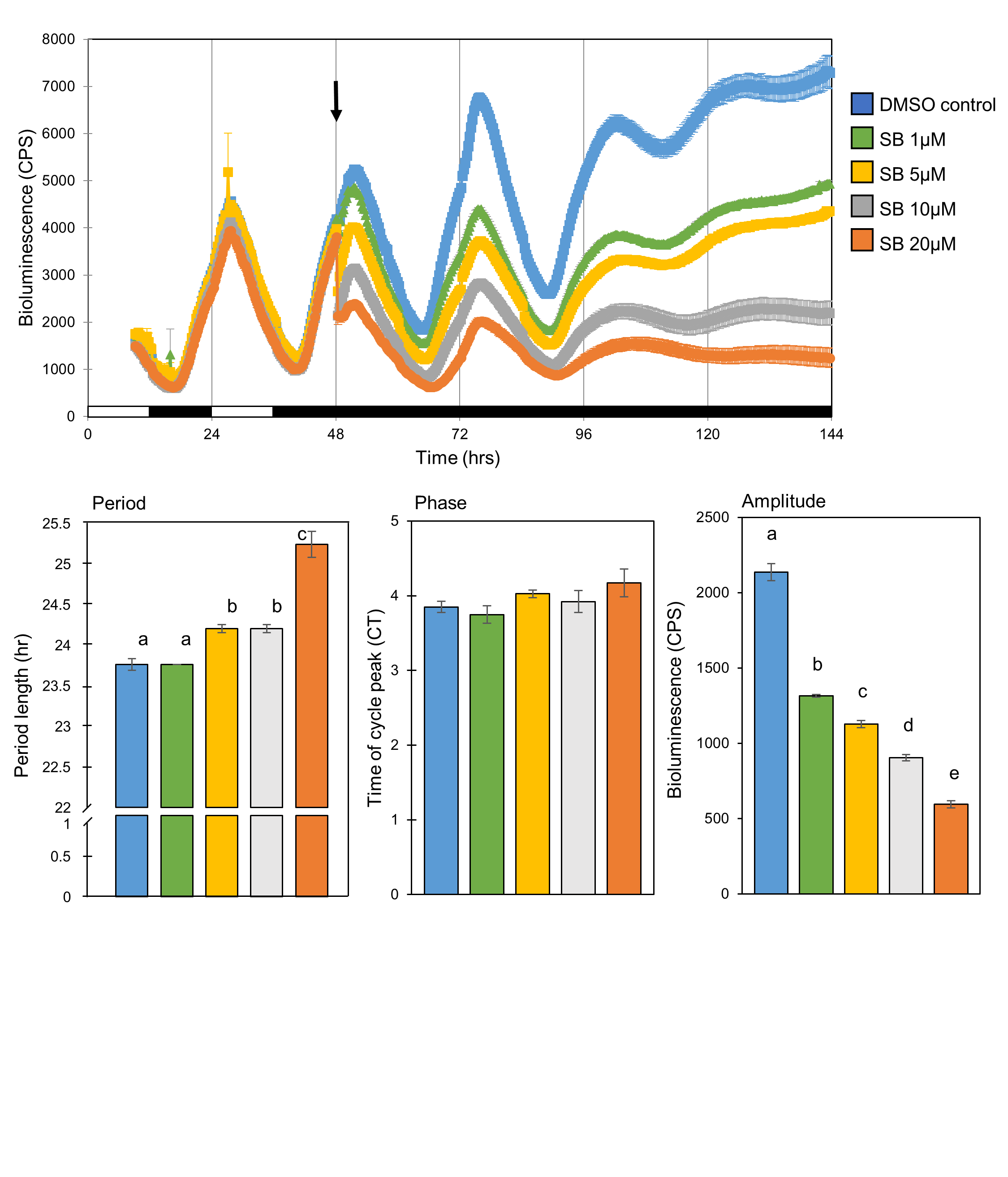

Supplement: S2 Fig — Rhythmic Per1b promotor activity in the zebrafish PAC-2 cell line was significantly altered by the addition of the TGF-β inhibitor SB-505124 in a dose-dependent manner in comparison to DMSO treated control (n = 8/group). Upper panel: bioluminescence is plotted on the y-axis and time (hours) on the x-axis. The horizontal bars represent the lighting conditions before and during sampling; white boxes represent light periods and black boxes represent dark periods. Lower panel: effects of inhibition on length, phase, and amplitude of Per1b promotor activity. Different letters represent statistically different values within each parameter (p<0.05, one-way ANOVA, Tukey’s test). Treatment led to a significant lengthening of the period of Per1b promotor activity (23.76±0, 24.2±05, 24.2±0.05, 25.24±0.16 hr for 1, 5, 10 and 20 μM, respectively, compared to 24.35±0.12 for the DMSO-treated), and reduction in the amplitude (1313.75.25±9.37, 1128±24.02, 903±20.26, 594.19±23.29 CPS for 1, 5, 10 and 20 μM, respectively, compared to 2136.25±57.29 for the DMSO-treated control), but not to a significant phase delay (the time of the first peak after the cells were transferred to DD was at CT 3.74±0.12, 4.025±0.05, 3.92±1.5, 4.17±0.19 hr for 1, 5, 10 and 20 μM, respectively, compared to 3.85±0.07 for the DMSO-treated control). (TIF) [file pone.0199777.s002.TIF]
